# Tracing Quiescent Cancer Cells In Vivo

**DOI:** 10.3390/cancers16223822

**Published:** 2024-11-14

**Authors:** Moon Jong Kim

**Affiliations:** 1 Department of Life Science, Gachon University, Seongnam 13120, Republic of Korea; moonjongkim7@gachon.ac.kr; 2 Department of Health Science and Technology, GAIHST, Lee Gil Ya Cancer and Diabetes Institute, Incheon 21999, Republic of Korea

**Keywords:** quiescent cancer cells (QCCs), cancer recurrence, tracing QCCs, in vivo mouse model, minimal residual disease

## Abstract

QCCs have long gained significant interest as potential “seeds” for recurrent cancers. Clinical evidence suggests that a subset of cancer cells exits the cell cycle and enters a quiescent state following anti-cancer treatment. These microscopic-residual QCCs are extremely challenging to trace and detect within patients. Additionally, QCCs resist conventional anti-cancer therapies due to the lack of cell activity. Notably, upon the unknown environmental cues in unknown time points, sometimes decades later, QCCs can reactivate, triggering cancer relapse at primary or secondary sites. Currently, no targeted therapies or diagnostic tools exist for QCCs, and their molecular regulatory mechanisms remain largely unknown. The major challenge in understanding QCCs lies in the limited availability of human-relevant pre-clinical models that trace and collect QCCs in vivo. This review provides an overview of existing QCC tracing systems and analyzes their limitations. It also cautiously proposes potential improvements for tracing QCCs in vivo based on recent advancements in QCC studies and lineage-tracing techniques. Developing human-relevant and easily accessible in vivo tracing systems will be a crucial step in advancing QCC diagnostics and therapeutic strategies.

## Simple Summary

Quiescent cancer cells (QCCs) remain unconquered challenges in cancer treatment. Even after being declared cured, patients remain at risk of cancer recurrence or face overtreatment aimed at eliminating minimal residual QCCs. The lack of effective diagnostic and therapeutic options for QCCs stems from our incomplete understanding of these cells. This knowledge gap is largely due to the scarcity or limitations of preclinical models, particularly those that enable the tracing of QCCs in vivo. By reviewing current QCC tracing tools and their limitations, this review aims to explore more advanced systems for tracing QCCs in vivo.

## 1. Introductions to Quiescent Cancer Cells

Cancer cell quiescence, also known as cancer cell dormancy, has been recognized in clinical practice for over a century [1,2,3,4]. Numerous clinicians and cancer researchers have observed and documented this phenomenon, which refers to cancer cells entering a state of quiescence—characteristically, inactive cells in the G0 phase of the cell cycle, marked by low Ki67 expression, reduced mRNA expression, or the de novo expression of cell cycle inhibitory proteins such as p27 [5,6,7,8,9,10].

**Figure 1:**
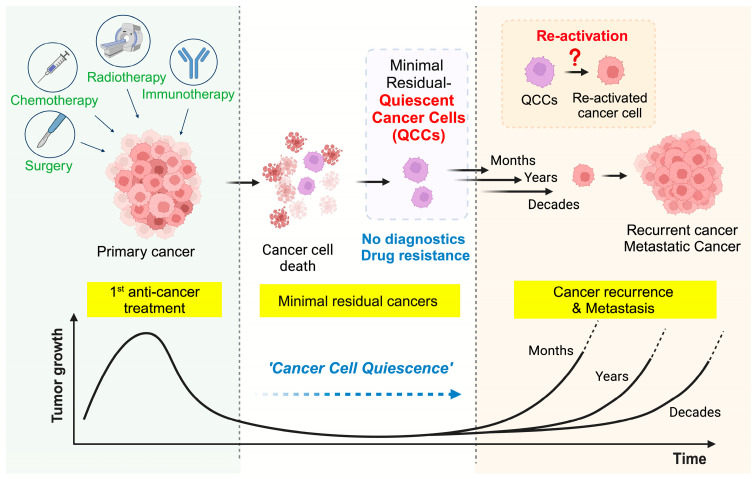
Overview of quiescent cancer cells: Seeds for recurrent cancer. After cancer treatment, most cancer cells are eliminated, but some cancer cells survive by entering an inactive state (cell cycle: G0 phase), also known as quiescence. These small QCCs may remain in the primary tumor site or circulate through the bloodstream (disseminate/circulating cancer cells), eventually being detected in other parts of the body. QCCs are believed to contribute to cancer relapse and metastasis by reactivating after weeks, years, or even decades.

In clinical settings, QCCs are often detected as subsets of circulating tumor cells (CTCs) in the bloodstream before and after cancer treatment [6,11,12]. Long-term in vitro and in vivo studies using cell lines and mouse models across various cancer types have consistently shown that QCCs exhibit resistance to anti-cancer drugs mainly targeting proliferating cells [13,14,15,16,17]. Given this, scientists have hypothesized that microscopic residual cancer, particularly QCCs, remains in the body following chemotherapy and may eventually reactivate, serving as “seeds” of cancer recurrence (Figure 1).

Notably, genetic analyses of recurrent cancers reveal that they originate from the initial tumors, suggesting that those quiescent cells can persist in the body for long periods—even years or decades—without obvious cellular activity. Based on these clinical observations, the concept of “minimal residual QCCs” (also referred to as minimal residual disease, or MRD, in hematologic malignancies) remaining quiescent and potentially reactivating to drive cancer recurrence has become widely accepted in cancer research [18,19].

However, it is important to note that this hypothesis has not yet been conclusively demonstrated in vivo, largely due to the lack of reliable models for tracing QCCs. Despite extensive research efforts, the precise mechanisms regulating QCCs remain elusive. This knowledge gap is exacerbated not only by the lack of appropriate in vivo preclinical QCC models but also by the variability in QCC collections derived from various xenograft experiments and patient samples. For instance, while large numbers of CTCs are detected in patient blood samples, only a small subset of these cells may actually lead to recurrence [2,20]. The majority of circulating cancer cells, which lack proliferative activity and eventually perish, may not represent the true seeds of cancer recurrence. Consequently, it remains unclear which circulating QCCs are the true drivers of recurrence in human patients, and mechanisms identified from whole CTC fractions may not accurately reflect the biology of QCCs.

Additionally, recent studies have identified other potential sources of MRD, including drug-resistant persistent cells, senescent cells, and polyploid large cancer cells [21,22,23,24]. However, it remains unclear whether these MRD-derived cells share characteristics with QCCs or represent distinct cell populations, highlighting the need for further investigation.

Currently, there are no specific biomarkers, accurate diagnostic methods, or effective strategies for targeting minimal residual QCCs [20,25]. To advance QCC diagnostics and therapeutic strategies, the development of new tracing methods for QCCs is a prerequisite. These new systems should represent the heterogeneity of human cancers, be easily applicable, and enable the in vivo tracing of QCCs in various states—active, quiescent, reactivated, and CTC conditions. This review first provides an overview of the current knowledge and existing markers/tracing tools for QCCs. By analyzing the advantages and limitations of these existing systems, this review aims to propose the next generation of QCC tracing methods.

## 2. Current Knowledge of Quiescent Cancer Cells

### 2.1. Cellular Inactivity, Prolonged Survival, and Reactivity of QCCs

To trace QCCs, it is essential to understand the key characteristics that distinguish them from other cancer cell types, such as high-propagating, low-propagating, apoptotic, and senescent cancer cells [2,3]. These distinguishing features can also help re-establish the current, often vague, definitions of QCCs.

First, cellular inactivity is a hallmark feature of QCCs [2,3]. Cancer cell quiescence refers to a state in which cells become inactive, typically characterized by their entry into the G0 phase, completely exiting the cell cycle. In this quiescent state, QCCs do not undergo cell divisions, resulting in no increase in cell number. In cellular inactivity status, cancer cells exhibit reduced mRNA transcription/protein translation, including many key cell cycle genes such as CDKs, cyclins, and cytokinesis-related genes [5,6,7,8,26]. Importantly, cellular inactivity could be confusing when distinguishing the QCC cells from apoptotic cells and senescent cells [19]. Thus, additional characteristics and markers are needed to define accurate QCCs.

Second, another defining feature of QCCs is their ability to survive in the patient’s body in an inactive state for extended periods, sometimes lasting years or even decades, without dividing [2,3]. Clinical observations suggest that QCCs maintain this prolonged cellular dormancy, allowing them to persist while evading immune detection [6,11,12]. However, it remains unclear how QCCs survive long-term without any cellular activity or whether they genuinely evade immune surveillance. The interaction between QCCs and the tumor microenvironment may contribute to their long-term survival and quiescence. For example, TGF-β and BMP signaling have been shown to maintain the quiescence of head and neck squamous cell carcinoma (HNSCC) cells and prostate cancer cells in bone marrow [27,28]. However, whether these signals are also involved in the long-term latency of QCCs remains uncertain. Many questions regarding this aspect of QCCs remain unanswered, and identifying the key factors that enable their long-term survival could present crucial targets for therapeutic intervention for QCCs [25].

Third, the last key characteristic of QCCs is their capacity for reversible reactivation [2,3]. Despite their long quiescence, QCCs likely sustain minimal cellular activity that allows them to respond to future “reactivation” signals. Under specific, or still poorly understood, conditions and stimuli, QCCs can exit their quiescent state, re-enter the cell cycle, and proliferate to form new cancerous masses [3,13,26,27,28,29,30,31]. This reactivation potential positions QCCs as potential cancer-initiating cells, akin to cancer stem cells or propagating cells. Indeed, inhibiting quiescence-inducing signals, such as DYRK1A, or activating signals like IFN-gamma and granulocyte colony-stimulating factor (G-CSF) can trigger the reactivation of cancer cells in certain contexts [8,18,32,33,34]. While it remains challenging to predict which QCCs will reactivate and which will perish, it is clear that only QCCs with reactivation potential serve as the true “seeds” for cancer recurrence.

### 2.2. Drug Resistances of QCCs

QCCs are thought to resist existing anti-proliferative cancer drugs due to their lack of cellular activity. This hypothesis has been supported by multiple in vivo and in vitro studies across various cancers [13,14,15,16,17]. These studies commonly show that a higher proportion of cancer cells remain in a quiescent state rather than actively dividing after anti-cancer therapy, with reactivation occurring at later stages. For example, an elegant experiment in glioblastoma mouse models using a ganciclovir-induced ablation tool specific for the population of QCCs (Nestin-ΔTK-GFP) revealed that a small population of Nestin-positive quiescent glioma cells remains after chemotherapy and acts as propagating seeds for glioblastoma recurrence [14]. In this study, Chen et al. also showed that ganciclovir-induced specific ablation of those glioma QCCs suppresses the tumor recurrence of glioblastoma.

Recurrent cancers typically acquire resistance to initial treatments, limiting therapeutic options and making them more difficult to eliminate. The fact that recurrent cancers can originate from microscopic residual QCCs suggests that the drug resistance observed in recurrent cancers may stem from these escapers [3,21,35,36]. In xenograft models of colorectal cancer, melanoma, and glioblastoma, slow-cycling cancer cell populations marked by H2B-eGFP pulse-chase systems consistently demonstrated greater chemoresistance in QCCs compared to other cancer cell populations [13].

However, the precise mechanisms underlying QCCs’ drug resistance remain unclear. Is this resistance inherently present in certain subpopulations of the tumor, such as slow-cycling cells or QCCs? In other words, is resistance the result of these quiescent and drug-resistant cancer clones being selected during anti-cancer treatments? Or do only certain QCCs acquire resistance through unknown mechanisms and then reactivate? If the latter is true, critical questions arise regarding when and how these QCCs develop resistance to existing therapies. Currently, these questions remain experimentally unproven. Further research is needed to clarify the mechanisms by which QCCs acquire drug resistance.

### 2.3. Immune Evasion and Interaction with Immune System

Immune surveillance plays a crucial role in defending against cancer. QCCs are thought to possess immune evasion mechanisms that enable them to escape immune detection. This hypothesis is supported by clinical observations showing that QCCs can persist in vivo for extended periods. Although the specific immune evasion strategies of QCCs are not yet fully understood, recent studies have provided some insights. One notable experiment suggests a potential mechanism by which QCCs evade immune surveillance. Latency-competent cancer (LCC) cells, derived from lung and breast cancers and exhibiting similar characteristics to QCCs, have been shown to evade NK cell-mediated clearance by downregulating ULBP ligands during metastasis [37]. This downregulation of NK cell-binding ligands in LCCs is driven by the inhibition of Wnt signaling through SOX2/SOX9-mediated expression of DKK1, a Wnt inhibitor. Additionally, recent studies have proposed new immune evasion mechanisms of QCCs, particularly against T cell-mediated attacks. Pilar et al. demonstrated that quiescent breast cancer cells resist T cell attack by creating a hypoxic niche, which impairs T cell function [38]. Furthermore, Goddard et al. suggest that the immune evasion of disseminated QCCs may result from scarce interactions between T cells and QCCs [39].

The interaction between QCCs and immune cells could be a driving force that either keeps cancer cells in a quiescent state or reactivates them. Recent studies have shown that immune cells within the tumor microenvironment may contribute to micro-metastasis and cancer recurrence. For example, neutrophils are essential for the colonization of metastatic mammary gland cancer cells in the lungs [40]. Furthermore, neutrophils remodel the extracellular matrix during inflammation, which can awaken quiescent breast cancer cells in the lungs of mouse models [41].

While many studies have advanced our understanding of the interaction between QCCs and the immune system, significant knowledge gaps remain. One critical question is whether these interactions could contribute to QCCs acquiring drug resistance over time. Additionally, it is necessary to investigate whether these mechanisms are common across various cancer types or vary depending on the genetic profile of each cancer. Further research is needed to elucidate the intrinsic mechanisms of immune evasion and the interactions between QCCs and the immune system. Understanding these regulatory processes is essential for developing strategies to target residual quiescent cells and prevent cancer recurrence through anticancer immunotherapy.

### 2.4. Molecular Mechanisms Regulating Quiescent Cancer Cells

Exploring the regulatory mechanisms of QCCs has been ongoing for a long time, yet many aspects remain unclear due to the challenges of collecting and tracing these small and heterogeneous populations of cancer cells. Despite limitations, significant progress has been made by researchers utilizing a limited number of, albeit imperfect, in vivo QCC models and clinical samples. Current knowledge indicates that various molecular pathways and signals regulate the quiescent state of cancer cells. For instance, p38 MAPK, TGF-β, BMP, the DREAM complex, Wnt/β-catenin signaling, and various histone modifiers have been implicated in maintaining and reactivating QCCs under different cancer conditions [8,13,25,27,29,37,42,43]. Due to space limitations, this review does not include a description of these regulatory mechanisms and instead focuses on addressing and analyzing tracing tools for QCCs.

## 3. Current Markers and Tracing Tools for Quiescent Cancer Cells

Several technologies have been developed to label and trace quiescent cells, both in normal physiology and in stem cell research (e.g., quiescent stem cells). These techniques were soon adapted for labeling QCCs. To date, the markers and tools used for identifying QCCs are listed in Table 1.

Each marker used for labeling QCCs has distinct differences and limitations. The first key distinction is whether the marker requires post-processing steps, such as fixation and immunostaining, or if it allows direct observation of cancer cells in their live state. Once QCCs are prepared, using live, unfixed cancer cells—rather than fixed and stained samples—is preferable for observing reversible reactivation and the dynamic processes of QCCs. The second distinction is whether the marker can be introduced at the cell line stage (requiring selection) or is available during all phases of cancer development (as in an autochthonous cancer model). In xenograft models, replicating the natural tumor microenvironment is challenging, and in syngeneic models, the use of advanced-stage cancer cells may result in missing critical windows of the cancer development process. An autochthonous cancer model developed in genetically engineered mouse models (GEMMs) allows researchers to better mimic human-relevant and heterogeneous cancer cell populations, compared to using selected homogeneous cancer cells for reporter insertion [55].

This section summarizes current markers and tracing tools for QCCs and analyzes their characteristics, including advantages and limitations. In the later part of this review, recent insightful findings using in vivo traceable systems for QCCs will be highlighted.

### 3.1. Negative Cell Proliferation Markers

The absence of proliferation has long been recognized as a hallmark of quiescent cells. Consequently, cells that do not express proliferation markers, such as Ki67 and PCNA, have been used to identify quiescent status. Indeed, Ki67 and PCNA are widely utilized in pathology analyses to predict cancer activity and prognosis.

However, it is important to note that Ki67- or PCNA-negative cells can exist in both the G0 phase (cell cycle exit) and the G1 phase (where cells are in the cycle but temporarily not expressing these markers). This overlap can lead to confusion between these two states. Therefore, it is essential to confirm that marker-negative cells are indeed in the resting G0 phase without intermittently expressing cell cycle markers. Another limitation is that these markers can only be detected through staining in cells or tissues, which poses a significant inconvenience. Consequently, it is not possible to trace whether Ki67-negative cancer cells remain quiescent, reactivate, or undergo cell death.

One alternative approach involves creating Ki67-reporter cancer cell lines to trace QCCs. However, selected cell lines often fail to reflect the heterogeneity of cancer. Moreover, because this method involves negative selection—where Ki67-negative cells are potential QCCs—additional markers or analyses are required for accuracy. Notably, Ki67-RFP knock-in mice were generated [46]. Although this model has not yet been widely used in cancer research, it holds promise for in vivo tracing of QCCs, as the absence of RFP signals indicates Ki67 negativity. Furthermore, this approach can be combined with various GEMMs.

### 3.2. Low mRNA Content

Due to the reduced cellular activity in quiescent cells, low levels of RNA transcription or protein translation serve as indicators of their resting phase. Several studies have indeed identified low mRNA content as a marker for QCCs [7,8,9,10]. Specifically, in fluorescence-activated single-cell sorting (FACS), G0 cancer cells can be distinguished in the G0/G1 phase by measuring marker intensities that quantify mRNA levels, such as pyronin Y, a cationic dye that intercalates with RNA [7,8,9,10].

However, this total mRNA labeling method is limited to fixed cells and can only be applied using flow cytometry, making it unsuitable for in vivo tracing. Additionally, a reporter for low protein translation in quiescent cells has yet to be developed. Therefore, new markers are needed that can effectively indicate low mRNA content or low protein translation in live cancer cells.

### 3.3. Cell Cycle and G0 Status Reporters

Fucci2 is a combined cell cycle marker that labels cells in different phases with distinct fluorescence (G1-red, S-yellow, G2/M-green), while G0-phase cells are indirectly marked by the absence of fluorescence [47]. As a result, G0 cells can be distinguished under a microscope or by FACS, though this process is challenging and not easily applicable to in vivo tracing. Also, this approach requires the selection process of generating reporter-inserted stable cancer cells.

DHB-mVenus (CDK2 sensor) distinguishes between G0/G1 phase cells and S/G2-M phase cells based on the nuclear localization of the CDK2 reporter [49]. DHB-Venus, a yellow fluorescent protein fused to DNA helicase B, includes four CDK2 phosphorylation sites within the nuclear localization signal region. In the G0/G1 phase, when CDK2 activity is low, fluorescence is observed in the nucleus; in the S/G2-M phase, when CDK2 activity is high, fluorescence appears in both the cytoplasm and nucleus. Since this reporter system cannot differentiate between the G0 and G1 phases, QCCs can only be identified by continuously tracing cancer cells that exhibit mVenus signals in the nucleus. Additionally, due to its dependence on the cellular localization of the fluorescent protein, this system is not widely used for in vivo tracing of QCCs.

mVenus-p27K(-) has recently emerged as one of the most commonly used markers for quiescent cells [5,38,48,56]. p27, a CDK inhibitor similar to p21 and p57, exhibits increased expression during the resting phase [5]. This reporter was developed to analyze cells in the G0/G1 phase (primarily G0) and has been widely utilized in vivo to label QCCs across various cancer models, including syngeneic models of murine mammary gland tumors and xenografts of human breast cancers. However, it remains to be verified whether p27 effectively marks QCCs across all cancer types and mutations. Additionally, like other reporter systems, this approach necessitates the generation of stable cancer cell lines, which may limit the heterogeneity of cancer cells. While combining it with GEMMs could enhance its applicability, it also requires considerable time and effort to develop these mouse models. Relevant studies employing this reporter system are discussed in the later part of this review.

### 3.4. Long-Term Labeling Retentions

Tracing long-term labeling retention cells (LRC; also referred to as LCCs, used interchangeably) is based on the principle that inactive cells do not divide [57,58]. In this approach, a constant amount of a chemical or protein reporter (such as histone-fused eGFP) is expressed as a marker, allowing it to persist within the cell for an extended period. In actively dividing cells, the marker content diminishes and eventually disappears with each cell division, while non-dividing and resting cells retain the marker. Thus, this method enables researchers to trace LRCs, which share characteristics similar to those of QCCs. Indeed, various versions of these techniques have been employed in several studies to trace inactive resting cells, including quiescent stem cells and QCCs.

Inactive cells were initially labeled using DNA-intercalating reagents such as BrdU, EdU, IdU, and CldU to trace quiescent LRCs [57,59]. Subsequently, long-lasting protein reporters, such as H2B-GFP, gained popularity [58]. When using chemical labels, inactive resting cells can be detected in tissue samples after a considerable time following labeling. Furthermore, by combining different chemical markers (e.g., BrdU + EdU), researchers can trace the inactivity and reactivation processes of quiescent cells in vivo. However, this method has significant limitations. Fixed samples are required, necessitating intensive histological work, and transcriptome/proteomics analysis is not feasible in fixed cells. Additionally, results are only available at the end of the experiment, which demands significant time and effort.

Therefore, doxycycline-inducible Tet ON-H2B-GFP offers the advantage of easy application in both cell lines and mice [58]. Also, this approach provides strong signal intensity and longevity, enabling live cell tracing in vivo. However, importantly, instances of reporter leakage have been reported both officially and unofficially [60], and even minor expression imperfections can compromise the pulse-chase methodology that traces quiescent cells through fluorescence intensity.

Another approach for tracing quiescent LRCs involves using live cell staining dyes [26,51,52,53]. Since these dyes penetrate living cells, they enable the direct study of heterogeneous cancer cell populations, unlike other experiments that require cell line selection. Several studies have investigated QCCs using various live cell stains. However, the non-fixed nature of the dye system poses risks, including the potential for the dye to transfer to neighboring cells or to originate from dead cells or cellular debris. Additionally, maintaining a strong signal can be challenging, making it difficult to utilize this approach directly for tracing living organisms. Despite these limitations, I personally expect that the live cell dye system still holds promise for future experiments using heterogeneous patient samples.

### 3.5. Limitations of Current QCC Tracing Tools

Despite the availability of various markers for QCCs, significant limitations remain in their application. Currently, the analysis of QCCs often requires additional methods, such as marker staining, reporter cell line selection, or specialized detection techniques, which present clear challenges for detecting and tracing QCCs in vivo.

## 4. Current Studies of In Vivo QCC Tracing Systems

This section overviews key studies that have traced QCCs in in vivo cancer models, including recent research. By examining the pros and cons of the in vivo tracing systems used in these studies, this review aims to cautiously predict the future direction of next-generation QCC tracing systems.

### 4.1. Using Pre-Selected Slow-Cycling Cancer Cells

#### 4.1.1. Pre-Selected Cancer Cells with Xenograft

The earliest significant discoveries regarding QCCs were largely made using xenograft models of pre-selected cancer cells. Although injecting selected human cancer cells into immunosuppressed mice has its limitations, this approach provides an accessible model, especially when suitable GEMM mouse models for tracing QCCs are unavailable. Consequently, xenograft models have been widely used in basic research to explore the mechanisms driving cancer cell quiescence and reactivation.

For instance, Bragado et al. used a xenograft model with GFP-labeled HEp3-GFP cells derived from HNSCC [27]. Quiescent cell populations collected from different environments (e.g., lung: LU-HEp3, bone marrow: BM-HEp3) demonstrated that cancer cell dormancy in the bone marrow is regulated by the TGF-β-p38 axis. In contrast, in the lung, a permissive environment with low TGF-β levels allowed QCCs to reactivate, leading to tumor growth and metastasis. This in vivo study was groundbreaking, providing the first molecular-level insight into how the microenvironment influences cancer quiescence and reactivation, revealing the presence of both inhibitory and permissive environments. However, the authors focused on pre-revealed signaling pathways (TGF-β and p38) and did not employ unbiased methodologies (e.g., RNA-seq) to discover new regulation mechanisms. In another study, Baldominos et al. used selected LCC cells (similar to QCCs) derived from breast cancer (HCC1954-LCC) and lung cancer (H2087-LCC) cell lines in xenograft models [37]. They reported the first immune evasion mechanism where circulating tumor cells (CTCs) secrete DKK1, which inhibits Wnt-ULBP ligands, enabling QCCs to evade natural killer (NK) cell attacks.

However, these xenograft models face significant limitations, as they rely on pre-selected certain cancer cells. Furthermore, using human cancer cells in immunosuppressed mice raises crucial questions about how well these findings translate to immunocompetent humans.

#### 4.1.2. Pre-Selected Cancer Cells with Syngeneic Models

The use of pre-selected slow-cycling cancer cells in syngeneic mouse models, derived from the same species, allows for the study of QCCs within an intact immune environment. This approach provides more accurate insights compared to xenograft models. Additionally, syngeneic models are easier to handle than genetic mouse models and offer a shorter experimental timeline.

For example, Lawson et al. elegantly demonstrated that the endosteal niche, controlled by osteoclasts, is crucial for the reactivation of quiescent myeloma cells in a syngeneic mouse model using double-labeling systems [54]. They employed syngeneic 5TGM1-eGFP murine myeloma cells and utilized live-cell staining dyes, DiD and CMDil, along with intravital two-photon microscopy to trace the in vivo reactivation process. QCCs were identified by the long retention of DiD dye, while the second dye allowed them to distinguish between long-term quiescent populations and reactivated cells.

Recently, in vivo syngeneic mouse models have been actively used to identify the intrinsic signaling pathways that suppress the reactivation of QCCs in response to interferon and TGF-β [61]. Hu et al. conducted a CRISPR/Cas9-mediated unbiased screen using a QCC line (KPad1) derived from a lung cancer mouse model (KP: *Kras^LSL-G12D^; p53^flox/flox^*). In conjunction with xenografts of human-selected QCCs (H2087-LCC) and utilizing syngeneic mouse models, they identified TGF-beta-mediated STING signaling as a key mechanism for maintaining cancer cell quiescence [61].

Additionally, p27-mVenus was incorporated into preselected syngeneic cancer cells and used versatilely in syngeneic mice. The application of p27-mVenus will be discussed in more detail in later sections.

Models using syngeneic mice still have the limitation of relying on selected cell lines, which are homogeneous. Nevertheless, due to their easy accessibility and compatibility with other QCC labeling technologies, the syngeneic system is expected to remain a vital tool for tracing QCCs in future research.

### 4.2. Tracing QCCs in GEMMs: The Most Powerful and Human-Relevant Tools

As previously mentioned, combining QCC tracing tools with GEMMs is one of the most powerful and relevant ways to study QCCs in vivo. However, due to the resource-intensive and time-consuming nature of this approach, relatively few studies have utilized this approach. This section highlights key research that has successfully integrated GEMMs with QCC tracing systems (Table 2).

Two pivotal studies, conducted simultaneously using the MMTV-Her2+ breast cancer model, provide significant insights [62,63]. The MMTV-Her2+ model closely mirrors the progression of human Her2-overexpressing breast cancer, allowing researchers to observe tumor development up to the metastatic stage [64]. Since the metastatic spread of Her2+ mammary cancer is primarily confined to the lungs, it is relatively easy to identify metastatic Her2+ cells through Anti-Her2 staining. Both research groups made the surprising discovery that metastatic Her2+ breast cancer cells migrate to the lungs at an early stage, and these early disseminated cells become most of the seeds for future metastasis [62,63]. Further analysis revealed that Her2/progesterone receptor signaling and Wnt-dependent epithelial-mesenchymal transition (EMT) processes are essential for driving these early disseminated cancers.

If we could develop tracing tools based on QCC-specific markers for various types of cancers and integrate them with GEMMs, this could represent the best strategy for detecting and tracing QCCs. Also, identifying these QCC-specific markers for diagnostics is one of the ultimate goals of QCC research. However, no definitive markers for identifying QCCs have been found yet. Instead, some studies have focused on markers that label remaining cancer cells after chemotherapy. While these markers are not perfect, they can identify residual cancer cells to some extent, allowing their fate to be traced using lineage-tracing mouse models. In fact, two studies used lineage-tracing models specific to post-chemotherapy residual cancer cells, combined with GEMMs. For example, in glioblastoma and basal cell carcinoma models, Nestin-positive and Lgr5-positive cells were used as markers for QCCs that persist after chemotherapy [14,65]. In both cases, GFP reporter systems were employed to trace these marker-positive cells, and genetic tools were used to induce targeted cell death (e.g., GCV-activated Nes-DTK-GFP or DTA-activated Lgr5-DTR-GFP). These studies confirmed that those persistent and residual slow-cycling cells act as seeds for cancer recurrence and resistance.

### 4.3. In Vivo Tracing QCCs Using mVenus-p27K(-)

Recently, studies utilizing mVenus-p27K(-) have gained significant attention, particularly in breast cancer metastasis models [38,56]. These studies often combine mVenus-p27K(-) with other QCC labeling systems, such as Tet-inducible H2B-GFP and Fucci, for more accurate tracing. While it remains to be confirmed whether p27, one of the cyclin-dependent kinase inhibitors (p21, p27, p57, etc.), can reliably mark QCCs in types other than breast cancer, it currently stands out as the most widely used reporter [5].

A notable recent study employed dual markers, mVenus-p27K(-) to label QCCs and Luc-Td-tomato to label all cancer cells, enabling researchers to trace cancer cells in vivo and distinguish QCCs from other cancer types [56]. This study utilized both xenograft and syngeneic mouse models, injecting human breast cancer cells (MDA-MB-231) and 4T1 mouse mammary gland cancer cells, respectively. Tissue samples were collected from all organs prone to metastasis to detect in vivo metastasis and investigate activation mechanisms. The findings revealed that IFN-gamma secreted by NK cells in the liver induced and maintained the quiescent phase in QCCs. Furthermore, the study discovered that breast QCCs could be reactivated by CXCL12 secretion from hepatic stellate cells, which occurred alongside hepatocyte damage and NK cell deactivation in the liver. This research is significant as it uncovered key molecular mechanisms governing the interactions between the tumor immune microenvironment and QCCs.

Another noteworthy study using the p27K(-) reporter system involved developing a combined system with T cells (Jedi-T cells) that can specifically kill cells expressing GFP, along with a new reporter system (PGK-p27K(-)-rtTA/TetOn-H2B-Tdtomato) to selectively label QCCs with tdTomato [38]. Intriguingly, the researchers found that QCCs (GFP+, tdTomato+) were more likely to evade T cell attacks. By performing spatial-single-cell RNA transcriptomics of the immune microenvironment surrounding these QCCs, using an mVenus-p27K(-) cell line, they discovered that an immunosuppressive niche forms around QCCs, protecting them from T cell-mediated destruction.

Additionally, another recent study explored the use of Lgr5-tdTomato, which marks the stemness of colorectal cancer (CRC) cells, alongside Fucci (for cell cycle labeling) and p27K(-) (a marker for QCCs) in a human-derived CRC organoid model [48]. The study examined the activity and quiescence of Lgr5-positive CRC cells both in vitro and in vivo, demonstrating that the interaction between cancer cells and the surrounding matrix is crucial for maintaining both quiescence and reactivation.

While these studies suggest that p27K(-) is a promising marker for QCCs in specific cancer types, it may not serve as a specific or universal marker for QCCs across various carcinomas as it could also label fully differentiated G0 cells or senescent cancer cells. Moreover, these studies predominantly relied on xenograft and syngeneic models, which use selected homogeneous cell lines and incomplete immune environments (e.g., PD-1 loss in Jedi mice), potentially limiting the relevance of the findings to human cancers. Further validation in more complex and clinically relevant models will be necessary to confirm these insights.

## 5. Next In Vivo Tracing Systems for QCCs

### 5.1. Ideal In Vivo Tracing Model for QCCs

The key considerations when developing an in vivo model for tracing QCCs are: first, how accurately the model reflects human cancer; second, whether QCCs can be reliably traced, identified, and isolated within the model; and third, how easily the model can be applied while minimizing the time required for preparation.

An ideal human-relevant cancer model should replicate the genetic characteristics of human cancer, including its progression through early, mid, and late stages, as well as the metastasis process and the surrounding tumor microenvironment. It should also capture cancer heterogeneity and support the application of clinically relevant anticancer treatments. In this context, the most comprehensive and human-relevant approach for QCC tracing would involve cancers that develop autochthonously in GEMMs [55].

However, only a few autochthonous cancer models adequately mimic the diversity and metastatic potential of human cancers. Notable examples include the MMTV-HER2 breast cancer model, the Kras*^LSL-G12D^*; p53*^flox/flox^* lung and pancreatic cancer model, and the Apc *^flox/flox^*; Kras*^LSL-G12D^*; p53*^flox/flox^* colon cancer model, which have been the focus of major studies [55]. These models take significant time to establish, and various QCC labeling systems must be integrated to effectively trace QCCs. As a result, relatively few studies have successfully traced QCCs in GEMMs in vivo.

Although current in vivo QCC tracing systems have limitations, recent advancements in CRISPR/Cas9-mediated gene editing via viral delivery systems present new opportunities to create models that enable faster and more efficient QCC tracing. Additionally, the ongoing discovery of new molecular markers for QCCs is likely to drive further advancements in QCC tracing models

### 5.2. New Technologies and Reporters for In Vivo QCC Tracing

Most current QCC studies rely on xenograft or syngeneic mouse models rather than GEMMs, primarily because of the extended time required to integrate QCC reporter systems into GEMMs and the challenges associated with tracing and isolating cancer cells that have metastasized and entered quiescence in target tissues. In GEMMs, metastatic cancer cells are not only scarce but also difficult to quantify and purify from tissues. As a result, no study has successfully isolated a small population of QCCs from GEMMs for single-cell sequencing, which typically requires at least 10,000 live cells.

Conversely, xenograft or syngeneic models offer the advantage of easily introducing various reporters, and by injecting large numbers of cancer cells, they reduce experimental variability and make it easier to isolate sufficient quantities of QCCs from metastatic sites. Consequently, most current research employs xenograft or syngeneic mice, with single-cell sequencing or spatial transcriptomics focusing on the surrounding microenvironment rather than on QCCs themselves.

Despite this, immunocompetent and autochthonous cancer models remain attractive options for QCC tracing due to their greater in vivo relevance, extended observation windows, and ability to model heterogeneous cancer development. This section highlights recently developed technologies and reporter systems that enhance the accessibility of QCC tracing in vivo, offering a glimpse into future advancements in biological models for studying quiescent cancer.

#### 5.2.1. Virus-Induced Genome Editing

**Figure 2:**
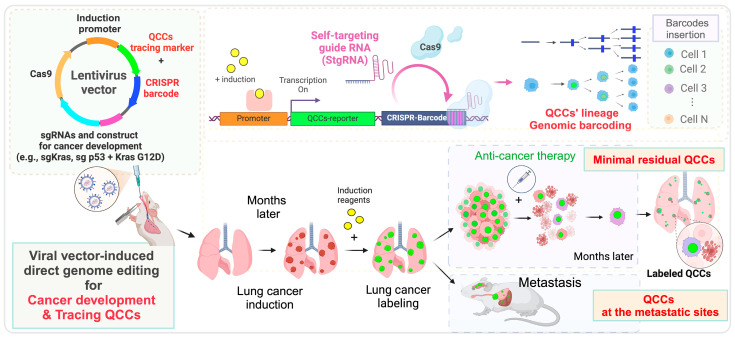
Potential advancements in next-generation in vivo QCC tracing system. A conceptual diagram illustrating the next-generation QCC tracing model for lung cancer. This advanced tool aims to induce cancer development using viral delivery vectors while simultaneously introducing QCC reporters into mice in vivo. Furthermore, when integrated with the newly developed CRISPR/Cas9-barcode lineage-tracing technology, this model is expected to overcome the limitations associated with the restricted marker repertoire of current QCC tracing systems.

Recent advancements in cancer research models have demonstrated that direct gene-editing techniques, such as CRISPR/Cas9 combined with viral delivery systems, significantly reduce the time and effort required to generate GEMM cancer models [66,67]. For instance, widely recognized models for lung, pancreas, liver cancers, and glioblastoma can now be produced through direct injections of CRISPR/Cas9 gene-editing constructs [66,67,68,69]. Initially, these techniques encountered challenges related to low or uneven efficiency, but ongoing research has validated their effectiveness, and increasingly efficient delivery systems continue to be developed. Incorporating a construct that simultaneously induces cancer development and includes reporters for QCC tracing would facilitate the rapid and efficient in vivo tracking of QCCs (Figure 2).

#### 5.2.2. CRISPR-Based Lineage Tracing

Current models that trace the fluorescent proteins of QCCs struggle to trace the reactivation of these cells. Tracing the transitions between activity and quiescent requires multiple reporters and additional the use of various chemical markers, such as BrdU and IdU. A recently designed CRISPR/Cas9-based barcode lineage-tracing system offers a solution to this limitation by expanding the repertoire of available reporters [59,70]. This allows for the tracing of QCC reactivation across multiple lineages of cancer cells. By performing genomic and transcriptomic analyses using CRISPR/Cas9 barcode systems alongside existing fluorescent protein-based QCC tracing tools, researchers may be able to trace the quiescent and reactivity of various QCC-descendant cancer cells (Figure 2).

#### 5.2.3. New Reporters Applicable to In Vivo QCC Tracing

Existing QCC tracing markers each have their strengths and weaknesses, and none are fully applicable across various cancer types. Consequently, there is an ongoing need to enhance current reporter systems or develop more precise markers for QCCs.

One unmet need in the field of QCC tracing is the lack of reporter systems that can directly observe mRNA/RNA translation in live cells. Developing a reporter capable of detecting low mRNA levels and translation activity would provide valuable insights. For instance, RGS2 (regulator of G-protein signaling 2), which regulates translation in lung cancer, was recently identified as both a marker and regulator of quiescent cancer, making it a promising tool for future QCC tracing [26].

Additionally, p57, a member of the cyclin-dependent kinase inhibitor family (which includes p27), has been identified as a marker for quiescent colorectal cancer [71]. When combined with other QCC tracing systems and CRISPR/Cas9 delivery technologies, these cell cycle-related markers, such as p27 and p57, could become more powerful tools for studying QCCs.

Lastly, the DREAM complex has been recognized as a key transcriptional repressor that controls cell quiescence, positioning it as a promising marker for QCC research [72]. While its roles in various cancers are not fully understood, recent studies have reported that the dissociation of the DREAM complex is essential for cancer progression in certain contexts [8,32,73]. Furthermore, p130, a component of the DREAM complex, exhibits dynamic localization—shuttling between the nucleus during the resting phase and the cytoplasm during the active phase. Developing a reporter system based on this behavior would be highly valuable. Further recent transcriptomic analyses involving the DREAM complex have been conducted, highlighting the potential for discovering new, effective reporter molecules [8,74].

## 6. Conclusions and Perspectives

Recent advances in QCC tracking systems and ongoing research efforts have significantly enhanced our understanding of these cells. However, these microscopic residual cancer cells, which exist in extremely small numbers within the body, remain largely unexplored. The diagnosing and tracing of QCCs continue to pose challenges, and the underlying mechanisms are not yet well understood.

To address this unmet need, it is essential to investigate the nature of QCCs in vivo, which fundamentally requires the establishment of models capable of tracing these elusive cells. The newly developed technologies and research approaches hold great promise for advancing in vivo QCC tracing methods. We look forward to leveraging these advancements to conduct research on QCCs and ultimately develop effective strategies for diagnosing and treating these cells.

## Figures and Tables

**Table 1:** Current markers and tracing tools for quiescent cancer cells.

Markers/ Tracing Tools	Marking Mechanism	Advantages	Limitations	Ref
Ki67-negative	Lack of cell proliferation marker, Ki67	1. Histological analysis 2. Ki67-RFP reporter mouse is available by negative selection	1. Labeling the mixed population of G0/G1 2. Need to fix/stain, making it impossible to trace the fate of QCCs in vivo	[44,45,46]
Pyronin Y	Low mRNA content	Direct indication for cellular inactivity	1. Need to fix/stain, making it impossible to trace the fate of QCCs in vivo 2. No clear distinction between G0 and G1 3. Not available for live cells	[7,8,9,10]
Fussi2 *	Marking G1/S/G2-M using multiple reporters	1. Available for live cells 2. Can be combined with graft models and GEMMs	1. Need to generate stable cell lines : Not reflect cancer cell heterogeneity 2. Negative selection: no color for G0 cells 3. Requires significant time and effort to generate and combine with GEMMs	[47,48]
DHB-mVenus	CDK2 activity sensors	Available for live cells	1. Need to generate stable cell lines : Not reflect cancer cell heterogeneity 2. Labeling the mixed population of G0/G1	[8,49]
mVenus-p27K(-) *	Reporter for cell cycle inhibitor, p27	1. Available for live cells 2. It can be combined with graft models and GEMMs	1. Need to generate stable cell lines : Not reflect cancer cell heterogeneity 2. Potential incompleteness of the p27 marker for QCC across different types of cancer 3. Requires significant time and effort to generate and combine with GEMMs	[5,38,50]
Live cell dye *	Tracing long-term labeling retention cells (=QCCs) after live-cell dye staining	1. Available for live cells 2. Selection is not required and can maintain the heterogeneity of cancer	1. In vivo systems are partially available 2. No clear distinction between G0 and other stages 3. Weak signal intensity during the tracing	[26,51,52,53,54]
Induceble * H2B-GFP	Long-term labeling retentions for QCCs using histone-fused GFP	1. Available for live cells 2. It can be combined with graft models and GEMMs	1. Need to generate stable cell lines : Not reflect cancer cell heterogeneity 2. No clear distinction between G0 and other stages 3. Requires significant time and effort to generate and combine with GEMMs	[13,16]

* Used in vivo systems, including xenograft, syngeneic, and GEMMs (genetically engineered mouse models).

**Table 2:** Recent studies utilizing in vivo QCC tracing systems.

In Vivo Model Type	In Vivo Tracing Tools for QCCs	QCCs Markers	Cancer Context	Limitations	Ref
Xenograft	GFP-labeled HEp3 cells, derived from QCCs in xenograft mouse model using head and neck squamous cell carcinoma (HNSCC)	GFP+ cell	HNSCC - lung and bone marrow metastasis	1. Homogenous selected cancer cell line 2. Immunocompromised mouse model 3. Human cancer + mouse tumor environment	[27]
Xenograft/ Syngeneic mouse model	Latency-competent cancer cells (LCC), derived from QCCs in metastatic sites of xenograft mouse model H2087 (lung cancer)-LCC HCC1954 (breast cancer)-LCC KPad1 (Kras*^LSL-G12D^*; p53*^flox/flox^* murine lung cancer model)	GFP+ and luciferase (TK-GFP-luciferase inserted)	Human lung/ breast cancer and murine lung cancer - various metastatic site	1. Homogenous selected cancer cell line 2. Immunocompromised mouse model 3. Human cancer + mouse tumor environment	[37,61]
Syngeneic mouse model	5TGM1-eGFP derived from murine myeloma cells with live-cell staining dyes (DiD and CMDil)	eGFP+ and dye+ cells	Murine myeloma - bone marrow metastasis	1. Homogenous selected cancer cell line 2. Used fully developed cancer: Omitted the process of invasion, intravasation	[54]
GEMMs	MMTV-Her2 MMTV-Her2-CFP (Murine mammary gland cancer model)	Her2+ stained or CFP+ cells	Murine mammary gland cancer - lung metastasis	Establishing a mouse model requires significant time and effort	[62,63]
Xenograft/ Syngeneic mouse model	MDA-MB-231 (Breast cancer) 4T1 (Mammary gland cancer) marked by mVenus-p27K(-) and Ubi-Luc-TdTomato	mVenus + Luc-Td-tomato + cells	Human breast cancer, murine mammary gland cancer - liver metastasis	1. Homogenous selected cancer cell line 2. Immunocompromised mouse model 3. Human cancer + mouse tumor environment	[56]
Syngeneic mouse model and GEMMs	4T07, EMT6, and D2A1 (Mammary gland cancer) marked by PGK-p27K(-)-rtTA /TetOn-H2B-Tdtomato and Jedi T cell mouse: Ptprc^a^; Tcrb^Ln1Bdb^; Tcra^Ln1Bdb^; H^2d^/J	mVenus + Td-tomato + cells	Murine TNBC mammary gland cancer	1. Homogenous selected cancer cell line 2. Artificial immune-modified model lacking PD1 and harbors GFP targeting TCRs	[38]

## Data Availability

Not applicable.
